# Effects of Radioactive Iodine on the Rat Thyroid's Function, Regeneration and Response to Goitrogens

**DOI:** 10.1038/bjc.1955.8

**Published:** 1955-03

**Authors:** I. Doniach, J. H. Logothetopoulos

## Abstract

**Images:**


### EFFECTS OF RADIOACTIVE IODINE ON THE RAT THYROID'S FUNCTION, REGENERATION AND RESPONSE TO GOITROGENS.

I. DONIACH AND J. H. LOGOTHETOPOULOS.

*From the Pathology Department, the Postgraduate Medical School of London.*

Received for publication January 18, 1955.

THE use of radioactive iodine ($I^{131}$) in tracer doses has proved extremely successful in experimental studies of the metabolism of iodine by the normal thyroid gland and together with the application of antithyroid drugs has led to considerable advances in thyroid physiology. However, there have been only few experimental investigations of thyroid function after treatment with moderately damaging doses of $I^{131}$ given in a dosage range equivalent to that used in the treatment of Graves' disease.

We report our findings below of tests carried out, after varying intervals of time, on rats injected intraperitoneally, at the age of 3 to 4 months with 30 microcuries of carrier free $NaI^{131}$. The average uptake by the thyroid gland was 20 per cent of the injected dose. We have tried to find the effect of this irradiation upon thyroid weight, follicle cell height and morphology, ability to concentrate iodide and bind iodine, to regenerate after partial thyroidectomy and to respond to a powerful goitrogen. We wanted to see if any functions were damaged preferentially. We also hoped that at least one of the tests might show an easily measured consistent deviation from the normal. We proposed, if successful, to apply this test in order to find what dosage of external X-irradiation was comparable in this biological effect with 30 $\mu$C of $I^{131}$.

### MATERIAL AND METHODS.

*Rats.*—(1) Black and white hooded Lister strain from a closed colony and (2) home bred albinos of mixed origin. All were fed on " Research " rat cubes and given greens twice a week.

*Propylthiouracil* (Williams & Co.).—The crystalline preparation was dissolved in water, 10 mg./1 ml. by the addition of drops of 40 per cent NaOH until a clear solution was obtained. Drops of 10 per cent HCl were then added until the pH was about 8·0 (B.D.H. universal indicator). Dilutions were made from this stock solution. Propylthiouracil-drinking water, 6 mg./10 ml. was made up twice weekly from fresh stock.

*Histological techniques.*—The thyroids were fixed, attached to the trachea, in Helly's fluid for 3 to 4 hours, washed overnight in tap water, dissected off the trachea and weighed. They were then dehydrated, cleared, embedded in wax and sectioned at 5$\mu$, cut horizontally in a central plane so as to include both lateral lobes and the isthmus. All were stained by haemalum and eosin and by the " tripas " periodic Schiff method of Pearse (1949). Many were stained by the Feulgen technique.

*Measurement of Radioactivity.*—The fixed and washed thyroids still attached to the trachea were each suspended at a fixed point in a standard $1\frac{1}{2} \times \frac{3}{4}$ inches screw-capped vial and counted in the multitube gamma Geiger Müller ring counter described by Veall and Baptista (1954). The count rates given by these thyroids represented organically bound $I^{131}$ only, since all inorganic iodide had been washed out of the tissues.

Digestion of thyroid tissue and plasma for counting (in order to estimate T/S ratios) was done in a mixture of equal parts 5 per cent NaOH and 95 per cent ethanol in an oven at 60° C. for a few hours. Each sample was digested in a total of 9 ml. fluid in a standard vial and counted as above.

*Measurement of thyroid plasma (T/S) iodide ratio.*—This was based on the principle and technique described by Vanderlaan and Vanderlaan (1947). Prior to injection of $I^{131}$, rats are pre-treated with a large dose of propylthiouracil, which prevents iodine from becoming organically bound. The measured radioactivity of the thyroid and blood is then entirely due to inorganic iodide. We injected the animals intraperitoneally with 10 mg. propylthiouracil in solution. Half an hour later each rat was injected subcutaneously with 50 $\mu$C $I^{131}$ in 1 ml. water. The rats were killed one hour after this by bleeding from the aorta under ether anaesthesia. Thyroid tissue, removed under the anaesthetic (after bleeding), was weighed immediately on a torsion balance and put into 9 ml. digest mixture in a standard vial. The average weight removed was 17 mg. Blood, collected into an oxalated tube was centrifuged and 1 ml. plasma taken into a standard vial containing 8 ml. digest mixture. After digestion at 60° C. the radioactivity was measured. The thyroid plasma (T/S) iodide ratio of each rat was calculated from its thyroid count rate per gram of thyroid tissue divided by the count rate of 1 ml. of its plasma.

*Measurement of mean follicle cell height.*—H. and E. stained sections were placed on the moving stage of a microscope set up for measuring red cell diameters and the image projected on to paper at 1000 times magnification. Two long lines which crossed centrally at right angles were drawn on the paper. In any projected field the images of occasional follicles lay by chance with their largest diameters directly coincident with one of the lines on the paper. The heights of the two cells, in these particular follicles, which lay along the lines were recorded. The heights of 200 cells were measured in each thyroid gland.

### EXPERIMENTS AND RESULTS.

*Experiment I.*

Ten out of 20 male hooded rats were injected intraperitoneally at the age of $3\frac{1}{2}$ months with 30 $\mu$C $I^{131}$. They and the 10 controls were killed by bleeding under ether anaesthesia 3 months later, 2 hours after an intraperitoneal injection of 10 $\mu$C $I^{131}$. The thyroids were fixed and washed. Measurements were made of their radioactivity (organically bound $I^{131}$), of their weights and of their mean follicle cell heights.

The results summarized in Table IA show that even though the previously irradiated rats were slightly heavier in body weight than the controls, their thyroids were smaller, 20·9 mg. against 26·9 mg. ($P < 0{\cdot}01$). The 2-hour uptake of $I^{131}$ was not significantly different in the two groups. However, this "normal" uptake of the smaller irradiated glands was associated with an increased mean follicular cell height, 7·6$\mu$ in 2000 cells against 6·6$\mu$ in the controls ($P < 0{\cdot}001$). Microscopic examination showed an associated diminution in colloid in the irradiated glands (Fig. 1 and 2) whose nuclei presented a greater variation in size than the controls.

TABLE IA.—*Body Weight, Thyroid Weight, Iodine Uptake and Follicle Cell Height after $I^{131}$.*

| Number of rats. | Treatment. | Mean body weight (g.) + S. deviation of mean. | Mean thyroid weight (mg.) + S. deviation of mean. | *Thyroid uptake of $I^{131}$.* Mean activity in 1000 counts/min. + S. deviation of mean (10 $\mu$C $I^{131}$ 2 hrs. before death). | Mean thyroid follicle cell height in $\mu$ (200 cells measured in each rat). |
|---|---|---|---|---|---|
| 10 | Nil | 303±36 | 26·9±3·9 | 5·5±1·3 | 6·58 |
| 10 | 30 $\mu$C $I^{131}$ 3 months previously | 315±23 | 20·9±2·9 | 5·7±1·2 | 7·64 |

In confirmation of this thyroid loss of weight following 30 $\mu$C $I^{131}$ we give in Table IB data obtained from another (unpublished) experiment in which the rats were treated with colchicine 8 hours before they were killed. Male albino rats were killed in groups of 7 irradiated and 7 controls at intervals of 24, 78 and 132 days after receiving 30 $\mu$C $I^{131}$. The body weights of the irradiated rats killed after 24 days were higher than the controls, but the body weights of the irradiated and controls respectively, killed at 78 and at 132 days, were similar; body growth was normal in the irradiated rats in both groups. There is no statistically significant difference in the irradiated and control thyroid weights at 24 and at 78 days; but at 132 days the irradiated thyroids weighed only 18 mg. in contrast to the controls 28 mg. ($P < 0{\cdot}01$).

TABLE IB.—*Body and Thyroid Weight at Varied Times after $I^{131}$.*

| Time in days after 30 $\mu$C $I^{131}$ | Number of rats. | | Mean body weight (g.) + S. deviation of mean. | Mean thyroid weight (mg.) + S. deviation of mean. |
|---|---|---|---|---|
| 24 | Controls | 7 | 262±19·8 | 23±3·6 |
| | Irradiated | 7 | 318±21·6 | 26±4·6 |
| 78 | Controls | 7 | 368±23·4 | 27±4·2 |
| | Irradiated | 7 | 364±24·1 | 21±2·0 |
| 132 | Controls | 7 | 387±28·7 | 28±6·9 |
| | Irradiated | 7 | 391±41·0 | 18±1·4 |

*Experiment* 2.

The thyroid : plasma iodide ratio was determined in 20 male hooded rats, 10 of which had received 30 $\mu$C $I^{131}$ 3 months previously. These were siblings of the animals in Experiment I.

The results summarized in Table II show no significant difference in the T/S iodide ratio, 75 ± 13 in the irradiated and 70 ± 22 in the controls. The body weights were similar in the two groups and no different from those in Experiment I.

*Experiment* 3.

Having found in Experiment I that the irradiated thyroids gave a normal 2-hour uptake of $I^{131}$, we wondered if they would regenerate after hemithyroidec-

TABLE II.—*T/S ratio after* $I^{131}$.

| Number of rats. | Treatment. | Mean body weight (g.) + S. deviation of mean. | Mean throid/plasma iodide ratio + S. deviation of mean. |
|---|---|---|---|
| 10 | Nil | 308±23 | 70±22 |
| 10 | 30 $\mu$C $I^{131}$ 3½ months previously | 298±22 | 75±13 |

tomy as efficiently as controls and if such stimulated glands would show a normal uptake of $I^{131}$. Ten out of 20 female hooded rats were injected intraperitoneally at the age of 3½ months, with 30 $\mu$C $I^{131}$. Eleven weeks later, the left lobe and left half of the isthmus of the thyroid was removed at operation under ether anaesthesia from them and from the 10 irradiated controls. Nine days later all 20 rats were given an intraperitoneal injection of 10 $\mu$C $I^{131}$ and killed after 2½ hours. The residual thyroid lobe was removed, fixed in Helly, washed, its radioactivity measured, the gland weighed and finally embedded in paraffin wax and sectioned as in Experiment I. Four additional female hooded rats, irradiated 3 months previously with 30 $\mu$C $I^{131}$, not hemithyroidectomized, were given 10 $\mu$C $I^{131}$ 2½ hours before being killed and the $I^{131}$ uptake of their thyroids measured.

The results summarized in Table III confirm the finding in Experiment I that the irradiated thyroids are smaller than those of controls 3 months after administration of 30 $\mu$C $I^{131}$, 7·3 mg. as against 9·4 mg. Their 2½-hour uptake of $I^{131}$ was the same as that of the unirradiated controls. The residual lobe, 9 days after hemithyroidectomy, showed a 2½-hour uptake of $I^{131}$ similar to that of the whole gland of the non-thyroidectomized rats.

The histological findings in the residual lobes of the hemithyroidectomized rats were interesting. In both groups the follicle cells were taller and colloid less in quantity than normal. The degree of activity varied, being more marked in the central region of the lateral lobes, many of the follicle cells there contained

---

EXPLANATION OF PLATES.

FIG. 1.—Thyroid of control rat. × 40.

FIG. 2.—Thyroid of rat njected 3 months previously with 30 $\mu$C $I^{131}$ showing smaller follicles, taller epithelium and less colloid than the control. × 40.

FIG. 3.—Thyroid of control rat 9 days after hemithyroidectomy showing hypertrophied cells. × 465.

FIG. 4.—Thyroid of rat 9 days after hemithyroidectomy, injected 3 months previously with 30 $\mu$C $I^{131}$, showing larger cells than the non-radiated hemithyroidectomized thyroids (Fig. 3) and a greater variation in nuclear size. × 465.

FIG. 5.—Thyroid of non-irradiated rat at end of 10 days propylthiouracil showing a typical goitrogen induced hypertrophy; tall cells, markedly diminished colloid and a mitotic figure. × 465.

FIG. 6.—Thyroid of rat at end of 10 days propylthiouracil, injected 31 days previously with 30 $\mu$C $I^{131}$, showing a goitrogen induced hypertrophy with larger cells and a greater variation in nuclear size than in the thyroids of the non-irradiated propylthiouracil treated rats (Fig. 5). Abnormal mitoses are present. × 465.

FIG. 7.—Thyroid of rat at end of 10 days propylthiouracil, injected 91 days previously with 30 $\mu$C $I^{131}$, showing an even greater variation in nuclear size and scattered micronuclei. × 465.

FIG. 8.—Thyroid of rat at end of 10 days propylthiouracil, injected 130 days previously with 30 $\mu$C $I^{131}$, showing bizarre-shaped nuclei, marked irregularity in follicle shape and occasional cells with clumped pycnotic chromatin. × 465.

1

2

3

4

5

6

7

8

Doniach and Logothetopoulous.

TABLE III.—*Effect of Hemithyroidectomy on Thyroid Weight and Iodine Uptake after $I^{131}$.*

| Number of rats. | Treatment. | Mean body weight (g.) + S. deviation of mean. | Mean weight (mg.) of residual thyroid lobe + S. deviation of mean. | *Thyroid uptake of $I^{131}$.* Mean thyroid activity in 1000 counts/min. + S. deviation of mean, $2\frac{1}{2}$ hours after 10 $\mu$C $^{131}$. |
|---|---|---|---|---|
| 10 | Hemithyroidectomized 9 days previously. No $I^{131}$ | $206 \pm 18$ | $9 \cdot 4 \pm 0 \cdot 6$ | $5 \cdot 9 \pm 1 \cdot 7$ |
| 10 | 30 $\mu$C $I^{131}$ 3 months previously. Hemithyroidectomized 9 days previously | $213 \pm 15$ | $7 \cdot 3 \pm 1 \cdot 0$ | $5 \cdot 2 \pm 1 \cdot 2$ |
| 4 | 30 $\mu$C $I^{131}$ 3 months previously not hemithyroidectomized | $191 \pm 30$ | $15 \cdot 4 \pm 1 \cdot 2$ | $5 \cdot 5 \pm 2 \cdot 6$ |

intracytoplasmic colloid droplets. The follicle cells of the irradiated glands appeared larger and showed a greater variation in nuclear size than the unirradiated hemithyroidectomized controls (Fig. 3 and 4). Unusually large nuclei and occasional micronuclei were present in the irradiated glands. Additional changes seen only in the irradiated glands, especially centrally, were bizarre-shaped nuclei, examples of marked infolding of the nuclear membrane, multinucleated follicle cells containing two or more small nuclei, and degenerate nuclei whose chromatin was seen as pycnotic dots lying within the ghost of a nuclear membrane.

*Experiment* 4.

In our results so far, we had failed to detect any loss of ability of iodine uptake in the irradiated thyroids. We had found a decrease in thyroid weight and the development of nuclear anomalies under the stimulus of regeneration. We therefore designed the following experiment to see if irradiated glands would show a reduced ability to undergo hyperplasia induced by a standard short course of propylthiouracil. We were particularly interested to see the effect of increasing the time-interval after irradiation when applying the goitrogen.

We found in preliminary trials with varying strengths of propylthiouracil solution that " drinking water " containing 6 mg. propylthiouracil per 10 ml. was well tolerated and was effectively goitrogenic. We also found, by killing off pairs of rats at 2-day intervals, that their thyroids, on this régime, showed a rising mitotic index up to about 8 days, followed by a plateau lasting about 4 days and then a fall to practically nil by 24 days. We then set up a series of 5 groups of animals, totalling 90 male albinos $2\frac{1}{2}$ months old. Each group contained 6 controls, 6 rats injected intraperitoneally with 10 $\mu$C $I^{131}$ and 6 given 30 $\mu$C $I^{131}$. Except for the week following the injection of $I^{131}$ the 18 rats in each group were housed in a single large cage. The goitrogenic stimulus was a 10-day course of 6 mg. propylthiouracil per 10 ml. in place of drinking water following an initial subcutaneous injection of a solution of 10 mg. propylthiouracil. The propylthiouracil was started 3 days after the $I^{131}$ in Group (a), 21 days in Group (b),

48 days in Group (c), 81 days in Group (d) and 120 days in Group (e). After 10 days on the propylthiouracil the rats were killed by coal gas, their thyroids removed and fixed in Helly for 3½ hours, washed, weighed, embedded in wax and sectioned as in Experiment I. Mitoses were counted, using an oil-immersion objective, the whole width or length of each thyroid lobe being traversed. The number of mitoses per 5000 cells was counted in each gland making a total of 30,000 for the controls and 30,000 for the rats given 30 $\mu$C $I^{131}$ in each group. Only cells in which the nuclear membrane had disappeared were registered as dividing, so that the mitotic count was virtually one of metaphases and anaphases, prophases being mostly classified as resting cells. In our counting we alternated the slides from controls and irradiated rats so as to keep our criteria as constant as possible.

The results summarized in Table IV show a remarkably consistent average weight of goitre in the control rats of Groups (a) to (e) varying from 55·5 mg. to 61·3 mg. in spite of the variation in the rats' average body weight : 245·8 g. to 376·7 g. and mitotic index : 60–208 mitoses per 30,000 cells. The average weight of the thyroids of all the irradiated rats was less than the controls. The most striking failure to respond to the goitrogenic stimulus was seen in the 30 $\mu$C rats 120 days after the administration of the $I^{131}$, Group (e). The diminution in goitrogenic response was evident in the 10 $\mu$C rats (42·5 mg. average thyroid weight), but did not alter significantly during the four months under study. A similar inhibition of goitrogenic response was produced by 30 $\mu$C but this became increasingly effective after 48 days when the average thyroid weight was 36·8 mg. It was 31·2 mg. following the goitrogen at 81 days and only 25·5 mg. at 120 days.

Histological examination of the thyroids showed the classical changes of hypertrophy and hyperplasia induced by goitrogens. Follicles were increased in number, colloid was grossly reduced, follicle cells were increased in height to above 15$\mu$, many contained intracytoplasmic colloid droplets (Fig. 5). In addition to numerous mitoses, the nuclei in control glands showed a greater variation in size and shape than those of unstimulated thyroids and very occasional micronuclei. However, the nuclei of the irradiated glands (10 $\mu$C and 30 $\mu$C) showed an even more striking variation in size and shape. Micronuclei appeared more numerous. The anomalies seen in the regenerating hemithyroidectomized irradiated glands were present and all were more marked (Fig. 6, 7 and 8). At 120 days, when mitoses were reduced to a minimum, cell hypertrophy was still as much in evidence as in the control unirradiated glands. The follicles of the irradiated glands showed a gradual increase in cell size.

Normal and abnormal mitoses were present in all the irradiated glands. The latter consisted mainly of irregular arrangements of the chromosomes at metaphase and anaphase. Occasional cells were present containing clumps of scattered deeply staining chromatin, an appearance suggestive of death and the onset of degeneration of a dividing cell. Multinucleate follicle cells with small overlapping nuclei were common.

Groups (f) 7 controls and 7 irradiated and (g) 6 controls and 7 irradiated male albino rats have been added to Table IV from two separate (unpublished) experiments. Their treatment was similar to Group (e) except that the 14 animals in Group (f) were injected with colchicine 8 hours before they were killed. All confirm the markedly reduced goitrogenic response 4 months after 30 $\mu$C $I^{131}$. The mean thyroid weights were 28 and 32 mg. in the irradiated and 52 and 54 mg. in the unirradiated controls.

TABLE IV.—*Goitrogenic Response after* $I^{131}$.

| Time after $I^{131}$ injection at which propylthiouracil course was commenced. | Controls. | | | 10 μC $I^{131}$. | | 30 μC $I^{131}$. | | | Ratio of mitoses in 30 μC rat thyroids to controls. |
|---|---|---|---|---|---|---|---|---|---|
| | Mean body weight in g. + S. deviation of mean. | Mean thyroid weight in mg. + S. deviation of mean. | Mitoses counted in 30,000 cells. | Mean body weight in g. + S. deviation of mean. | Mean thyroid weight in mg. + S. deviation of mean. | Mean body weight in g. + S. deviation of mean. | Mean thyroid weight in mg. + S. deviation of mean. | Mitoses counted in 30,000 cells. | |
| 3 days (a) | 245·8±15·6 | 59·3± 7·8 | 184 | 242·5±20·9 | 42·5±6·5 | 225·8±18·0 | 35·8±4·4 | 88 | 0·48 |
| 21 days (b) | 279·2±33·9 | 55·5± 4·5 | 121 | 265·0±14·5 | 40·8±6·0 | 281·7±24·4 | 40·7±6·3 | 68 | 0·56 |
| 48 days (c) | 343·0±17·2 | 59·0±11·5 | 208 | 331·0±13·5 | 47·2±9·4 | 330·8±17·2 | 36·8±4·4 | 58 | 0·28 |
| 81 days (d) | 345·8±31·6 | 60·2±11·9 | 60 | 338·3±43·9 | 40·8±5·5 | 352·5±22·5 | 31·2±4·3 | 14 | 0·23 |
| 120 days (e) | 376·7±36·7 | 61·3±10·3 | 172 | 413·0±36·2 | 41·0±8·2 | 390·0±35·6 | 25·5±4·7 | 6 | 0·035 |
| 132 days (f) | 369·0±63·4 | 52·0±10·4 | 349 (colchicine) | — | — | 380·0±62·7 | 29·0±8·8 | 30 (colchicine) | 0·086 |
| 129 days (g) | 364·0±16·4 | 54·0± 7·1 | 112 | — | — | 418·0±19·4 | 32·0±3·6 | 35 | 0·31 |

The mitotic index was less in all the irradiated rats than in the controls. It was reduced to a half of that of the controls at 3 days and 21 days in the 30 $\mu$C groups and to less than a third at 48 days and at 81 days. After 4 months, namely, Groups (e), (f) and (g), the results varied. The mitotic index was reduced in all. This was most striking in (e) and (f) in which it was reduced to less than one-tenth of the controls. In Group (g) it was only reduced to one-third.

*Experiment* 5.

The goitrogenic response 4 months after irradiation was tested in a similar way to Experiment 4. Half the animals were given $I^{131}$ and half given external X-irradiation to the thyroid region by Dr. J. D. Abbatt. We are reporting the results in detail in a separate paper. However, we should like to note here that a similar degree of inhibition of goitrogenic response resulting from 30 $\mu$C $I^{131}$ was obtained with a dose of 1000 roentgens of 190 kV X rays to the thyroid. (The diameter of the X ray beam was 13 mm. and the dose rate at the thyroid gland, taken as 8 mm. deep, was 150 r/min.)

### DISCUSSION.

The impossibility of assessing accurately the radiation dosage to the thyroid from $I^{131}$ because of variation between follicles in iodine uptake and turnover, radiation cross-fire at the centres of the lobules and escape at the periphery has been discussed previously (Feller, Chaikoff, Taurog and Jones, 1949; Maloof, Dobyns and Vickery, 1952; Doniach, 1953). The dosage range to the thyroids in rats which absorbed 20 per cent of 30 $\mu$C $I^{131}$ was considered to lie between 3200 and 22,600 rads. (Doniach, 1953). The experimental findings reported above show that this irradiation produces a loss of thyroid weight which becomes more marked with time. The reduction in weight was definite after 3 months (Tables IA and IB). In previous experiments (Doniach 1950 and 1953) the thyroid weight was still further reduced 15 months after $I^{131}$. The loss of weight was not associated with any obvious loss of function. The animals grew normally, their smaller thyroids concentrated iodide normally (Experiment 2) and showed a 2-hour uptake of bound iodine quantitatively similar to the larger unirradiated glands. It would appear likely that the smaller irradiated thyroids maintain a euthyroid state by increased activity of their cells. This was borne out by the findings of an increased mean cell height and a diminution in colloid. On the other hand we expected to find that this hypertrophy would be associated with other evidence of a relative increase in circulating pituitary thyrotrophic hormone (T.S.H.). We hoped, therefore, to find an associated increase in T/S iodide ratio.

Our failure to do so suggests that this method was not sensitive enough to detect the increased thyroid activity manifested quite clearly in histological sections. The variation in T/S iodide ratio was large in the rats in any one group. Statistical evidence of an increase in iodide concentration might have been found if many more rats had been used.

The findings in Experiment 3 prove that even after hemithyroidectomy the residual lobe of the irradiated gland can still function adequately, i.e. bind iodine in a similar amount to a non-hemithyroidectomized irradiated animal or a hemithyroidectomized non-irradiated one. But histology shows that this is done at the expense of a very marked cell hypertrophy. Moreover, disturbing nuclear anomalies and degenerative cells are seen which suggest that the irradiated thyroid might be reaching the limit of its compensatory faculties.

The results of Experiment 4 bring out a number of major points. The irradiated thyroid cells are capable of full hypertrophy; in this experiment definitely in response to an increased blood level of T.S.H. following a 10-day course of a potent goitrogen. On the other hand the ability of the irradiated thyroid to undergo hyperplasia is reduced since histology showed a lowered mitotic index. Furthermore, this reduction in goitrogenic response becomes more marked with time. The non-stimulated irradiated thyroid glands at 4 months averaged 18 mg. (Table IB). The goitrogen stimulated irradiated thyroids at 4 months averaged 25·5 mg. (Table IV), Group (e), the stimulated controls 61·3. Presumably the increase in weight from 18 to 25·5 mg. was due partly to cell hypertrophy and to hyperaemia, and possibly to an increase in cell number since there were 6 mitoses counted in 30,000 cells. Irradiation may inhibit hyperplasia directly by the prevention of mitosis or may so damage cells that they break down after entering mitosis. The histological findings in Experiment 4 of abnormal resting and dividing nuclei are consistent with a radiation effect. Some of the appearances noted favour the likelihood of cell degeneration following an abnormal mitosis. We do not know why this radiation response of inhibition of hyperplasia increases with time. Most radiation experiments on plant and mammalian tissues have dealt with the action of radiation upon proliferating cells. This contrasts with the present experiment in which the irradiation was administered to a resting tissue stimulated to proliferate many weeks later. Though our findings suggest that the longer the time-interval following irradiation the more hazardous is cell division, we have no reason to assume that breakdown in division accounts entirely for the thyroid's inability to grow.

It is difficult to explain why thyroid cells should be either increasingly less able to survive mitosis or else become increasingly unresponsive to a mitotic stimulus with the passage of time after exposure to irradiation. Billen, Stapleton and Hollaender (1953) found that following the X-irradiation of resting *Escherichia coli* the apparently non-viable bacterial cells showed a capacity, limited in time, for normal respiratory activity. The authors postulated that this might have been effected by a limited reserve of enzymes whose reformation had been inhibited by the X-irradiation. We might postulate along these lines that irradiation diminishes the ability of the thyroid cells to reform hypothetical enzymes which are gradually used up in time by normal cell activity and are essential for division.

Skanse (1948) studied the effects of nondestructive doses of $I^{131}$ upon thyroid function. He used 5-day old cockerels primed with 3 daily injections of T.S.H. and tested the effects of 1, 10 and 50 $\mu$C$I^{131}$ upon thyroid growth, collection of $I^{131}$, response to thiouracil and to T.S.H. The calculated radiation dose to the thyroid was about 1700, 13,000 and 60,000 rep respectively for the 1, 10 and 50 $\mu$C. Normal thyroid growth was significantly inhibited by the larger doses. Ability to take up iodine was not altered in the 10 $\mu$C chicks, but was decreased in the 50 $\mu$C group. A 10-day course of thiouracil started 26 days after the $I^{131}$ produced an increase in thyroid weight in all groups, but the 50 $\mu$C group did not show any thyroid weight increase in response to a 10-day course of thiouracil started 38 days after the $I^{131}$.

Maloof, Dobyns and Vickery (1952) carried out concurrent functional and histological studies on rats injected with 1 to 300 $\mu$C $I^{131}$ at intervals ranging from 2 to 18 months after the $I^{131}$ administration. No impairment in body weight gain was noted in animals receiving 30,000 rep or less to the thyroid. There was a persistent increase in follicle cell height in all animals that had received a calculated maximum dose of 5800 rep or more to the thyroid. This was associated with a diminished thyroid gland weight but a normal ability to take up a tracer dose of $I^{131}$, 48 days after the initial irradiation. A 30-day course of thiouracil, instituted 64 days after the initial radiation showed a good goitrogenic response in the animals which had received 1800 rep to the thyroid, a diminished response in the 5800 and 30,000 rep-groups and no goitrogenic response at all in groups which had received 80,000 rep or more to the thyroid. The authors described bizarre nuclear changes in the follicle cells after irradiation, accentuated by thiouracil administration and noted the irradiated thyroids' retention of ability to undergo cellular hypertrophy. They thought that the failure of response to thiouracil suggested an impairment of cellular division.

Our results largely confirm these radiation effects following the appropriate dosage of $I^{131}$, in particular the reduction in thyroid weight, maintenance of normal body weight and thyroid ability to take up iodine, increase in follicle cell height and ability to hypertrophy, inhibition of ability to undergo hyperplasia and the formation of bizarre nuclear forms. We have extended Skanse's observation that time after irradiation enhances the lack of responsiveness to thiouracil and confirmed by mitotic counts the suggestion of Maloof *et al.* (1952) of an impairment of cell division.

Our findings show that the degree of goitrogenic response 4 or more months after irradiation of the thyroid is a sensitive and easily determined index of radiation damage. The test proved satisfactory for the quantitative comparison of the effects of external X rays with internal $I^{131}$ irradiation.

The other test which proved simple but less sensitive was the measurement of the post-irradiation loss of weight of the non-goitrogen stimulated thyroids. The mechanism is presumably due to the same hyperplasia inhibition noted above, except that here we are dealing with a failure of the normal growth and renewal in time of thyroid tissue rather than the failure to respond to exogenously applied growth stimulus. This finding suggests that given enough time these irradiated thyroids might undergo a complete atrophy. It follows that some patients successfully treated with $I^{131}$ for Graves' disease might nevertheless develop myxoedema due to post-irradiation thyroid atrophy many years after. The only function that we have found to be affected preferentially in rats by moderately damaging doses of $I^{131}$ appears to be concerned with cell renewal; secretory function of the gland as a whole appears unchanged.

The capacity to divide is not lost altogether since the atrophying thyroid following 30 $\mu$C $I^{131}$ grows occasional adenomata and when submitted to a prolonged goitrogenic stimulus may produce carcinomata (Doniach, 1953). The destructive dose of 100 $\mu$C $I^{131}$ almost totally eliminated this capacity to produce adenomata (Doniach, 1953). However, Goldberg and Chaikoff (1952) obtained 7 thyroid carcinomas in 25 rats each given 400 $\mu$C $I^{131}$ 18 months previously. Those post-irradiation cells which have retained the ability to undergo successful mitosis appear more liable than unirradiated cells to give rise to tumours when stimulated to division by a maintained rise in circulating thyrotrophic hormone.

SUMMARY.

Thyroid function tests were carried out on rats injected 3 months previously with 30 $\mu$C $I^{131}$, a dose considered comparable with that used in the treatment of Graves' disease. There was a loss of thyroid weight, an increase in follicle cell height, a normal T/S iodide ratio and a normal thyroid uptake of $I^{131}$. The residual lobe after hemithyroidectomy showed a similar $I^{131}$ uptake, a smaller mass, and a greater cell hypertrophy than non-irradiated hemithyroidectomized controls. The irradiated glands showed bizarre nuclear forms. A series of rats was killed at the end of a 10-day course of propylthiouracil administered at varying time-intervals after 10 and 30 $\mu$C $I^{131}$. The goitrogenic response was less in the irradiated than in non-irradiated controls and diminished considerably with time in the 30 $\mu$C group. This impaired goitrogenic response was associated with a marked reduction in mitosis; cell hypertrophy was not reduced. The findings of abnormal mitoses, micronuclei and other bizarre nuclear forms favour the hypothesis that part of the inability to undergo hyperplasia is due to a radiation effect on the nucleus. Measurement of the diminished goitrogenic response 4 months after 30 $\mu$C $I^{131}$ proved a sensitive test for radiation damage and was found applicable to the measurement of X-irradiation damage.

We are indebted to the Medical Research Council for a personal grant to one of us (J. H. L.). We are grateful to Alma Howard for advice on interpretation of the cytological changes after radiation. We thank J. G. Griffin for the sections, C. A. P. Graham for the photographs and Jennifer Strudwick for help with the calculations.

REFERENCES.

BILLEN, D., STAPLETON, G. E. AND HOLLAENDER, A.—(1953) *J. Bact.*, **65**, 131.
DONIACH, I.—(1950) *Brit. J. Cancer*, **4**, 223.—(1953) *Ibid.*, **7**, 181.
GOLDBERG, R. C. AND CHAIKOFF, I. L.—(1952) *Arch. Path.*, **53**, 22.
FELLER, D. D., CHAIKOFF, I. L., TAUROG, A. AND JONES, H. B.—(1949) *Endocrinology*, **45**, 464.
MALOOF, F., DOBYNS, B. M. AND VICKERY, A. L.—(1952) *Ibid.*, **50**, 612.
PEARSE, A. G. E.—(1949) *J. Path. Bact.*, **61**, 195.
SKANSE, B. N.—(1948) *J. clin. Endocrin.*, **8**, 707.
VANDERLAAN, J. E. AND VANDERLAAN, W. P.—(1947) *Endocrinology*, **40**, 403.
VEALL, N. AND BAPTISTA, A. M.—(1954) *Brit. J. Radiol.*, **27**, 198.


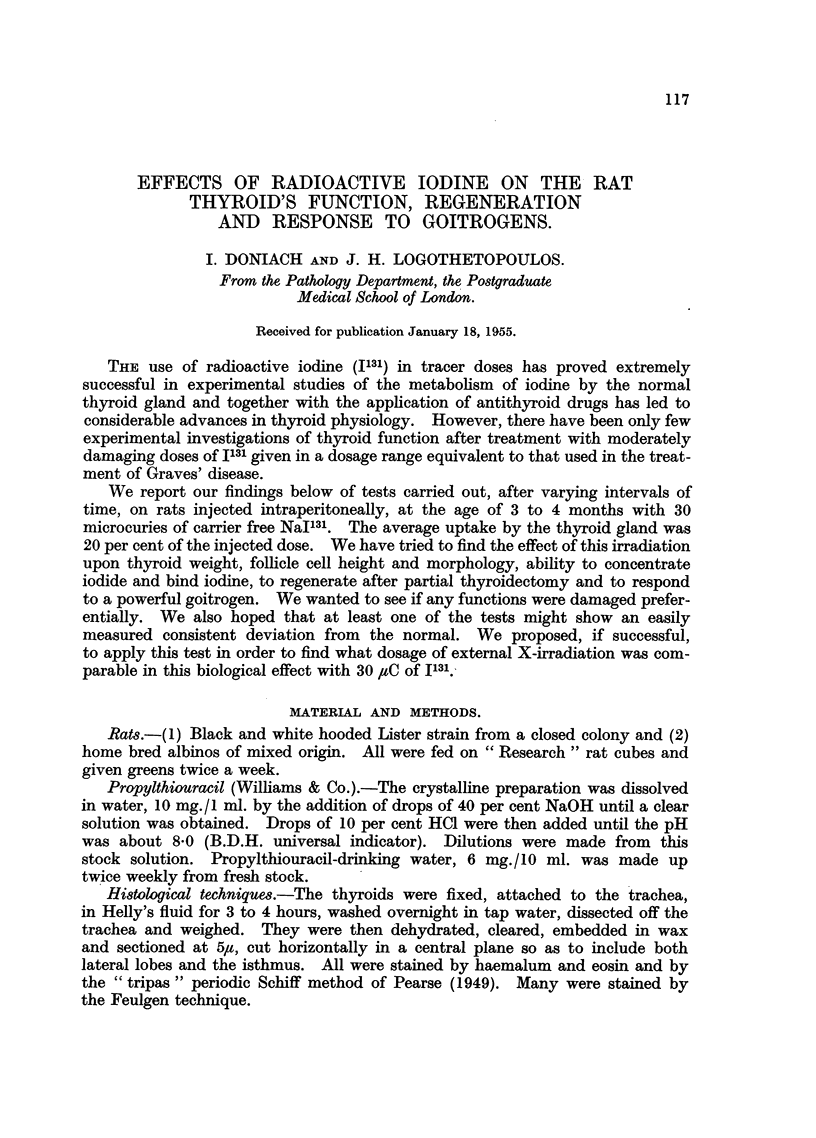

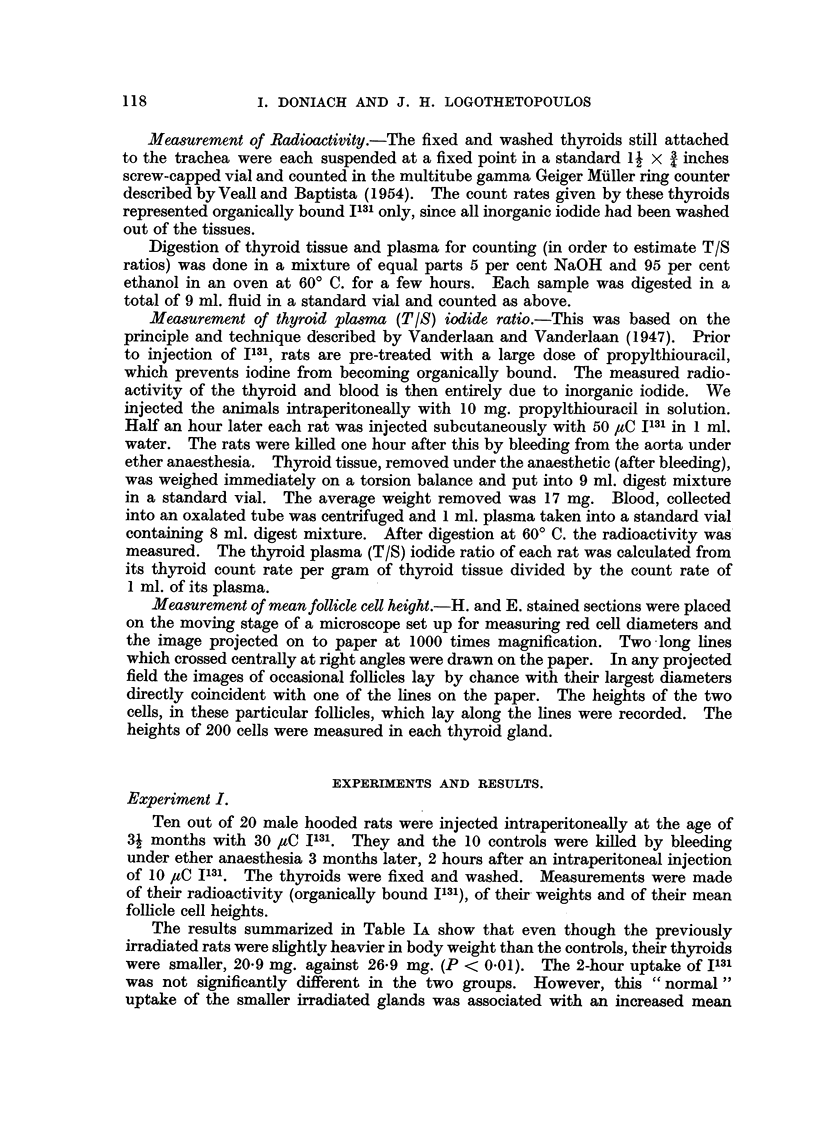

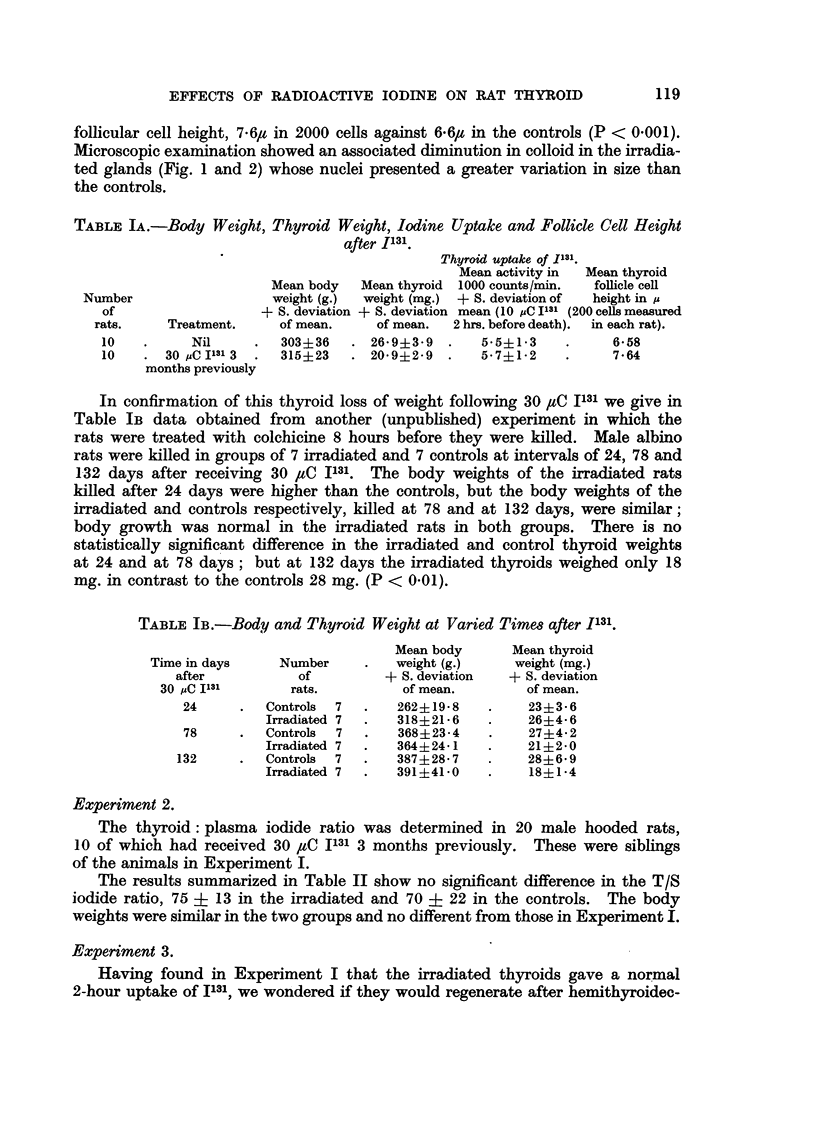

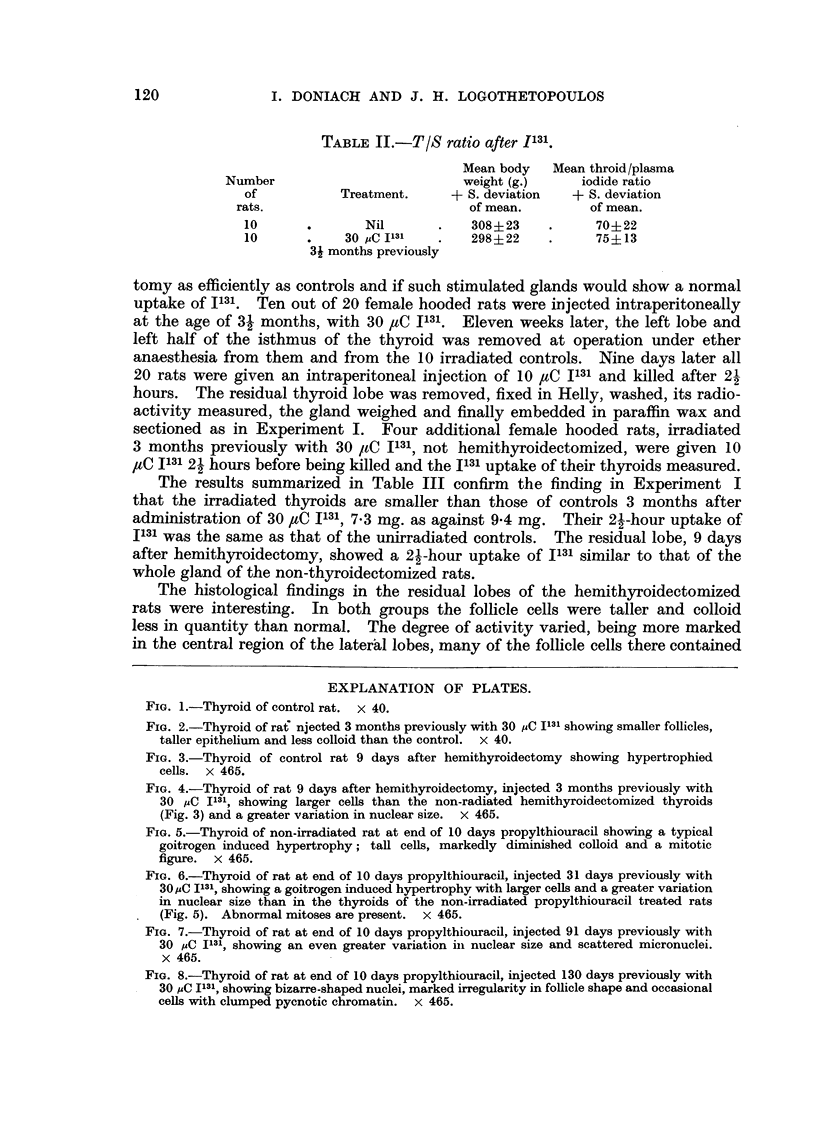

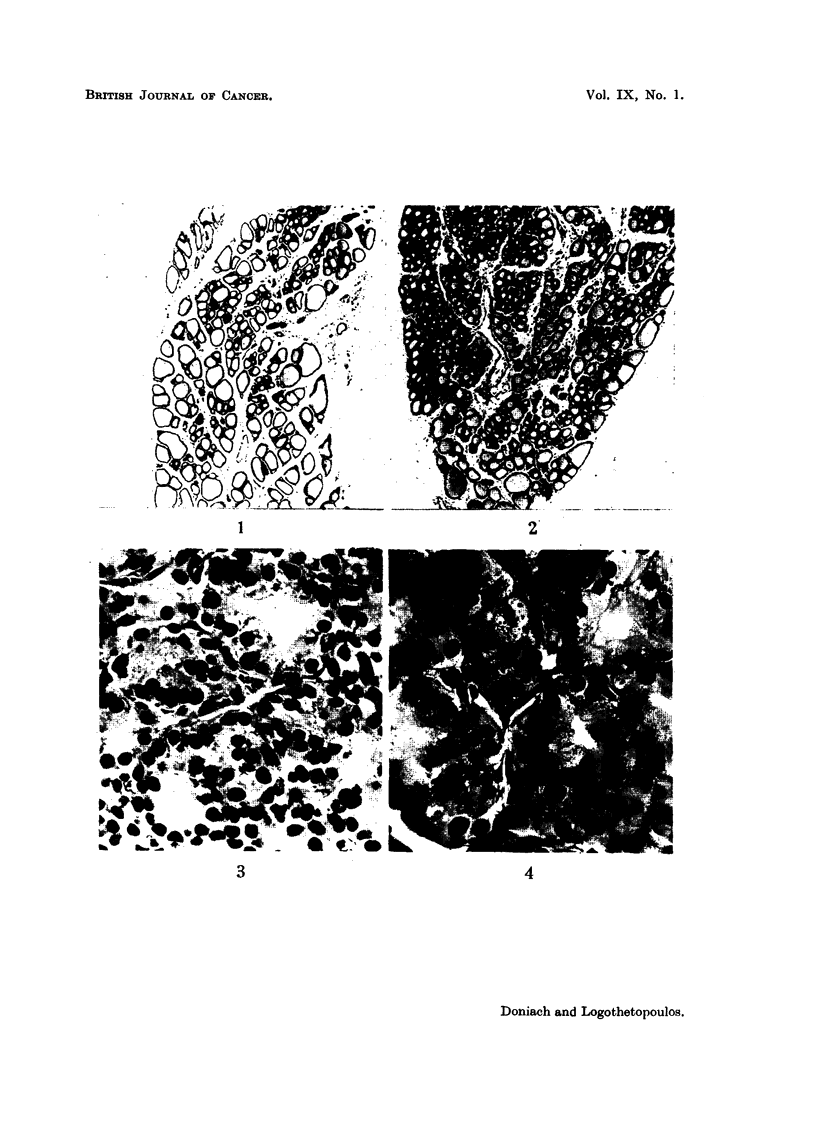

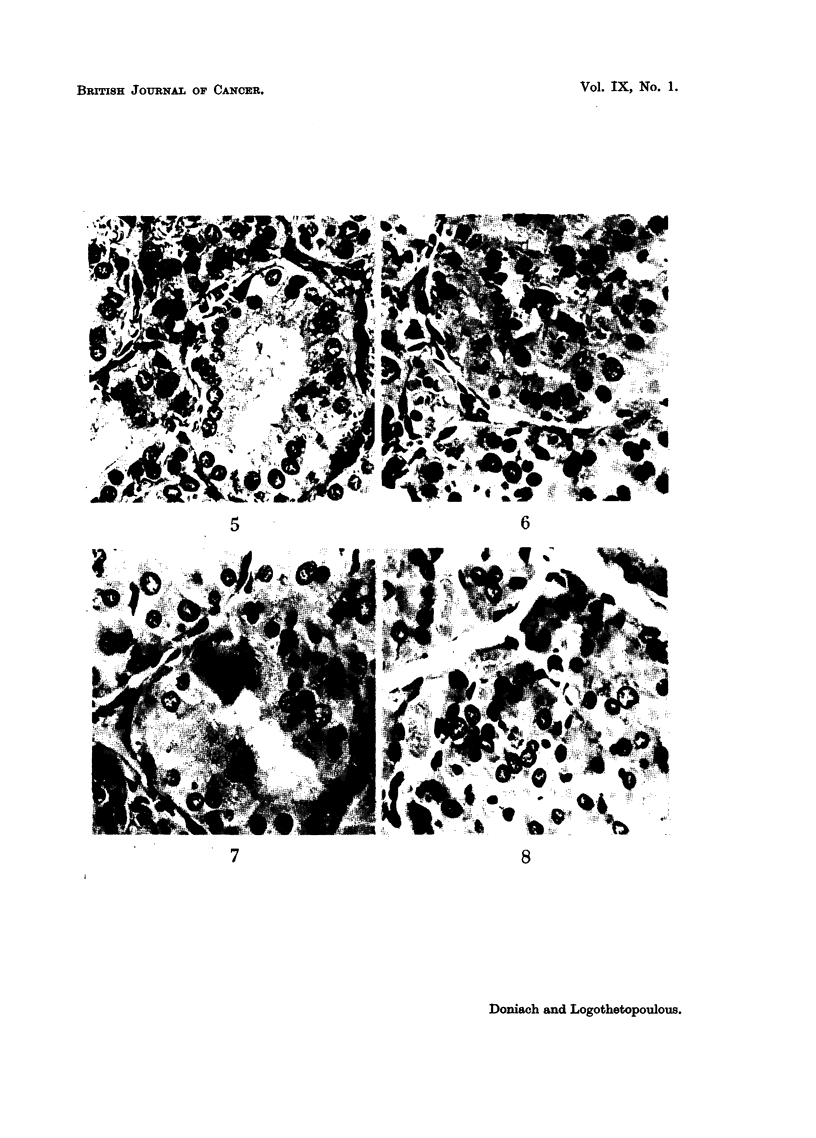

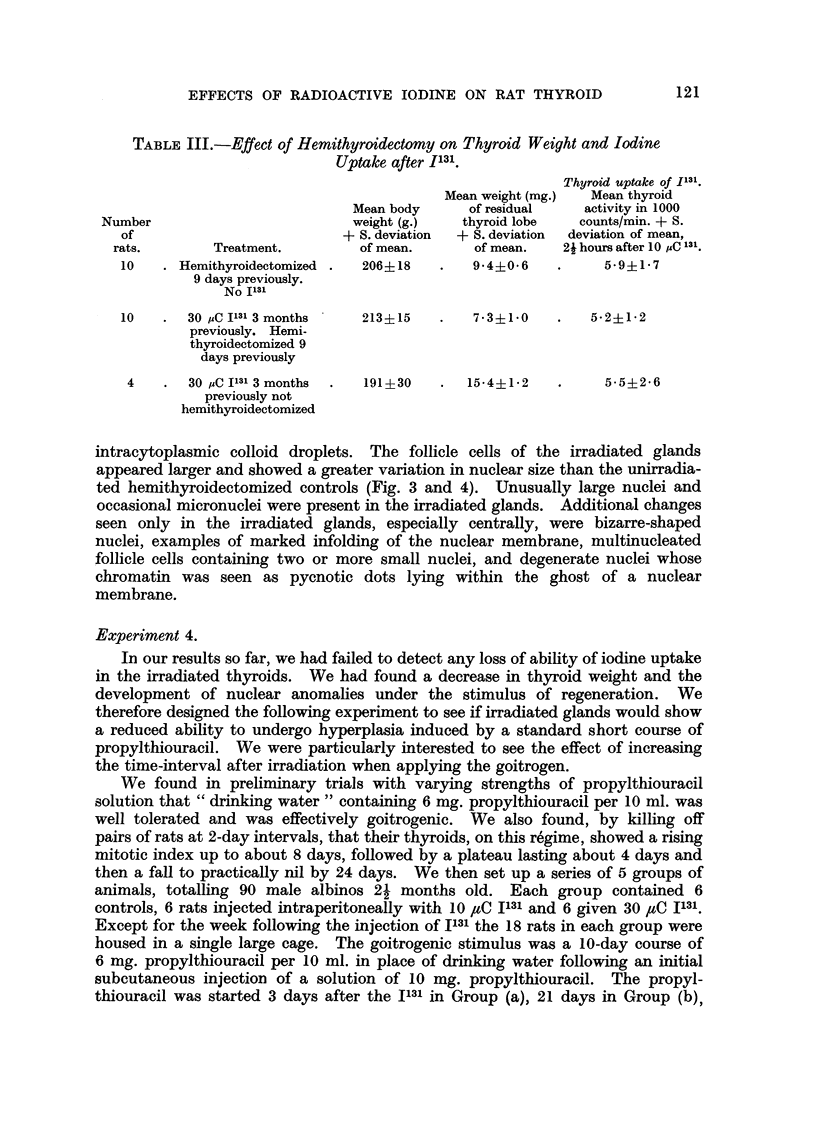

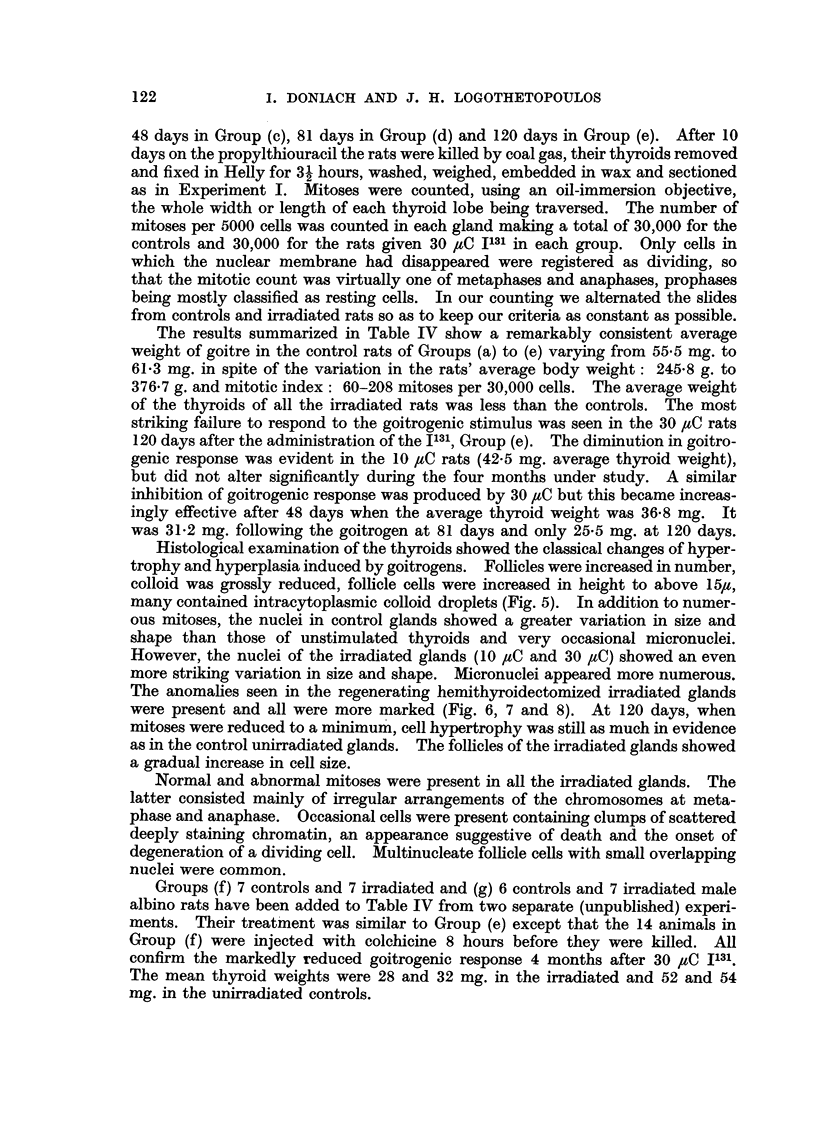

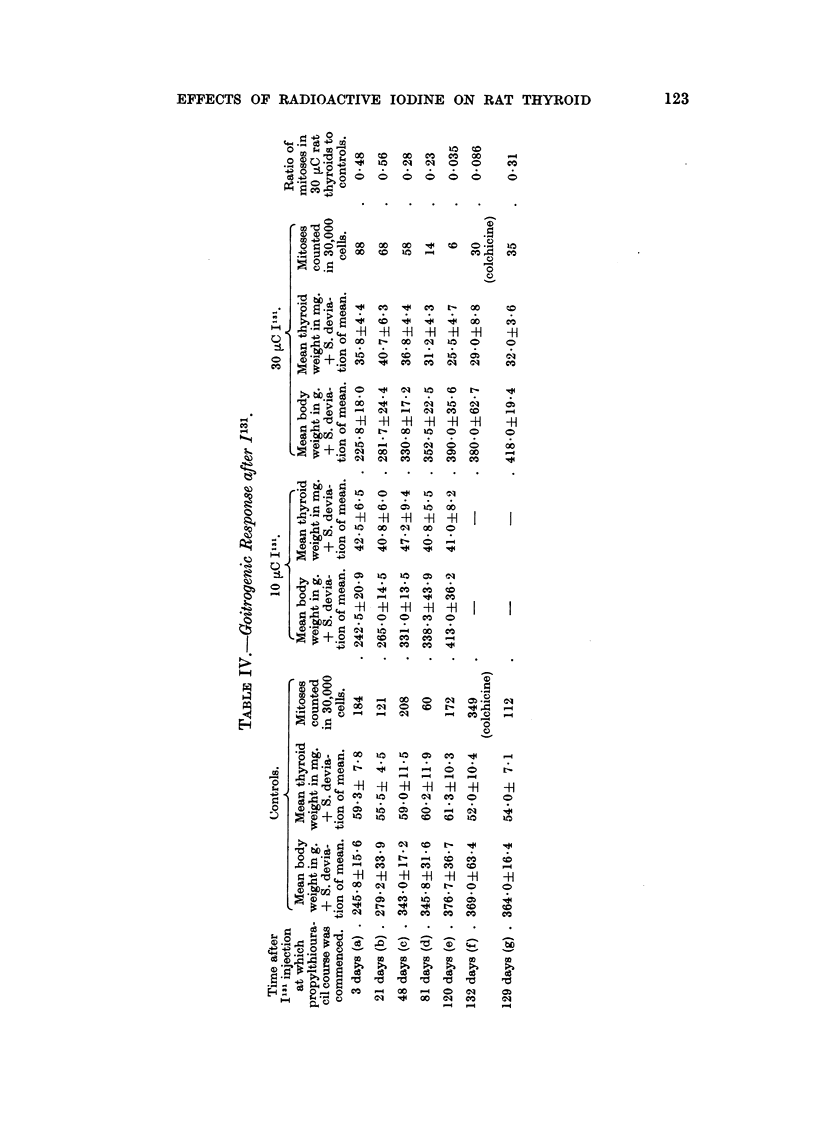

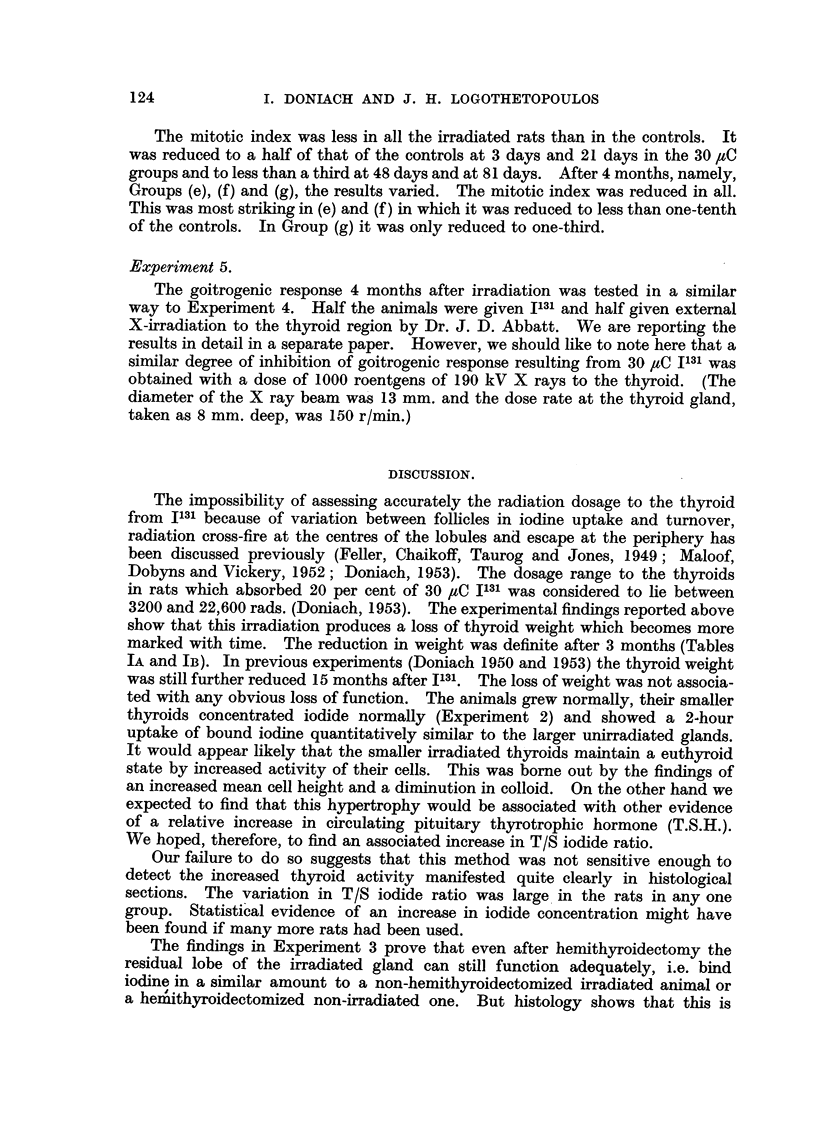

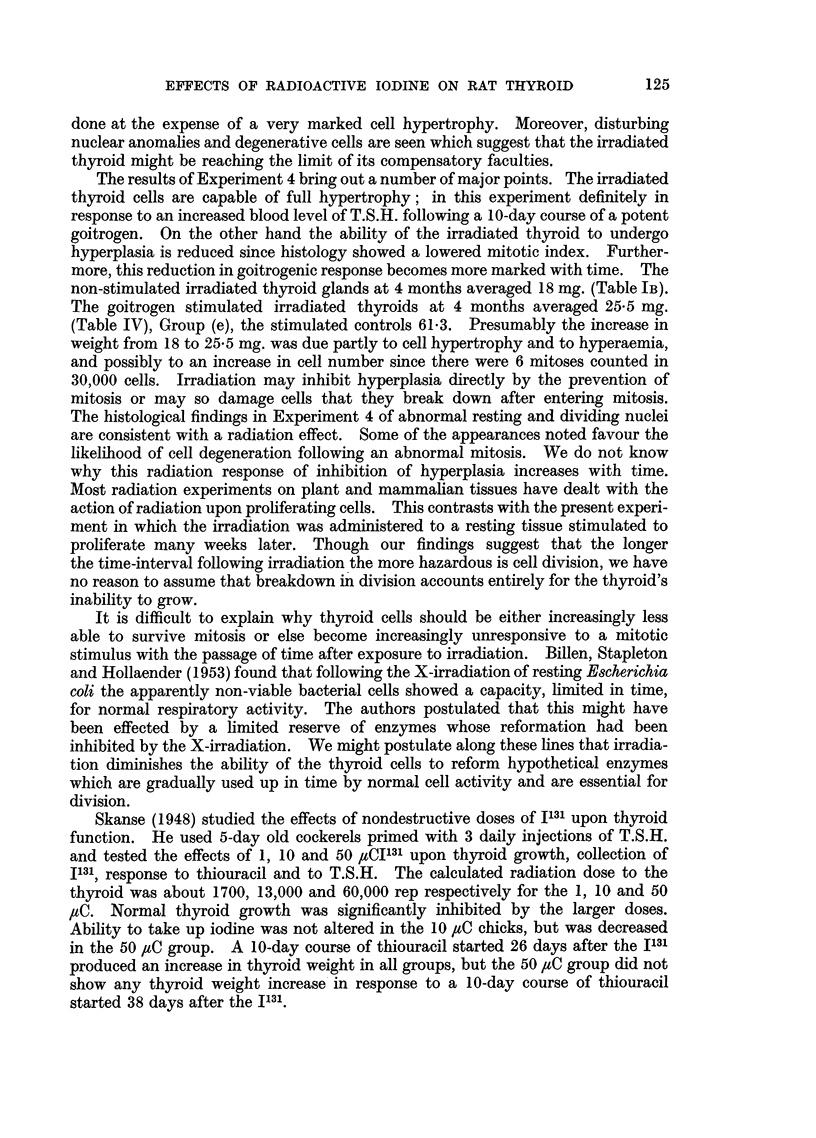

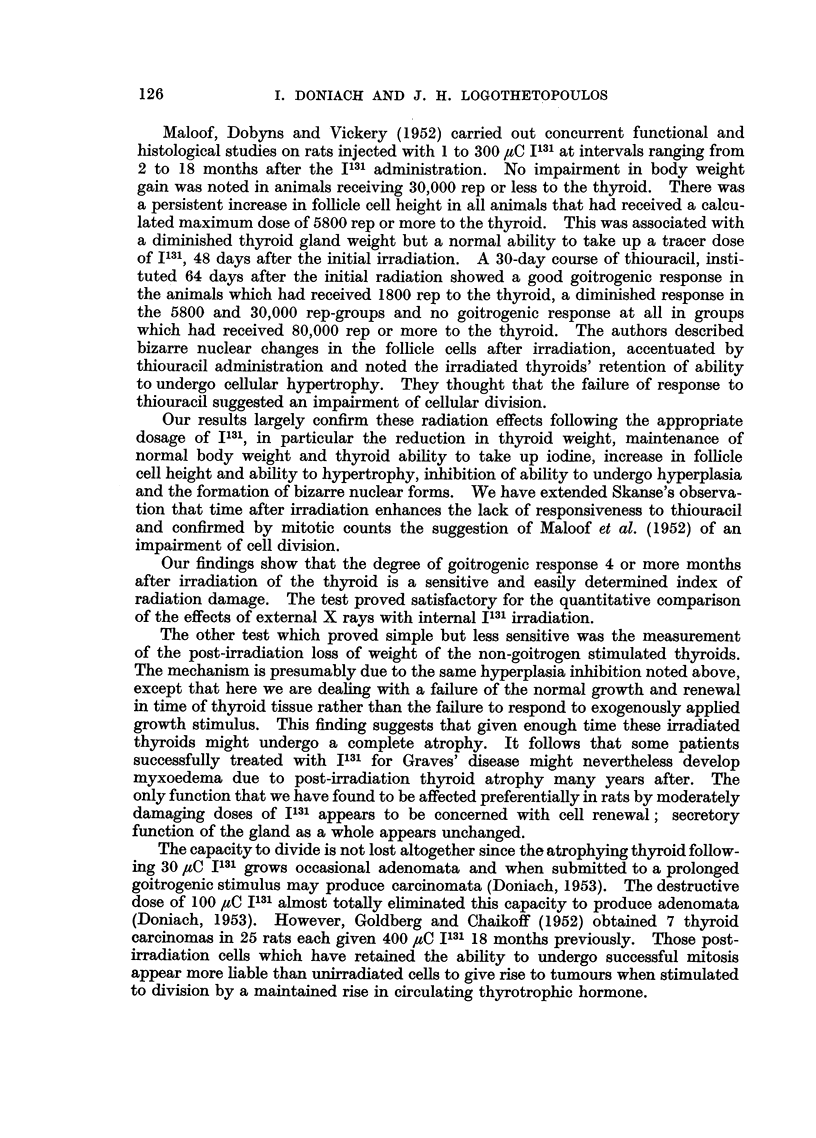

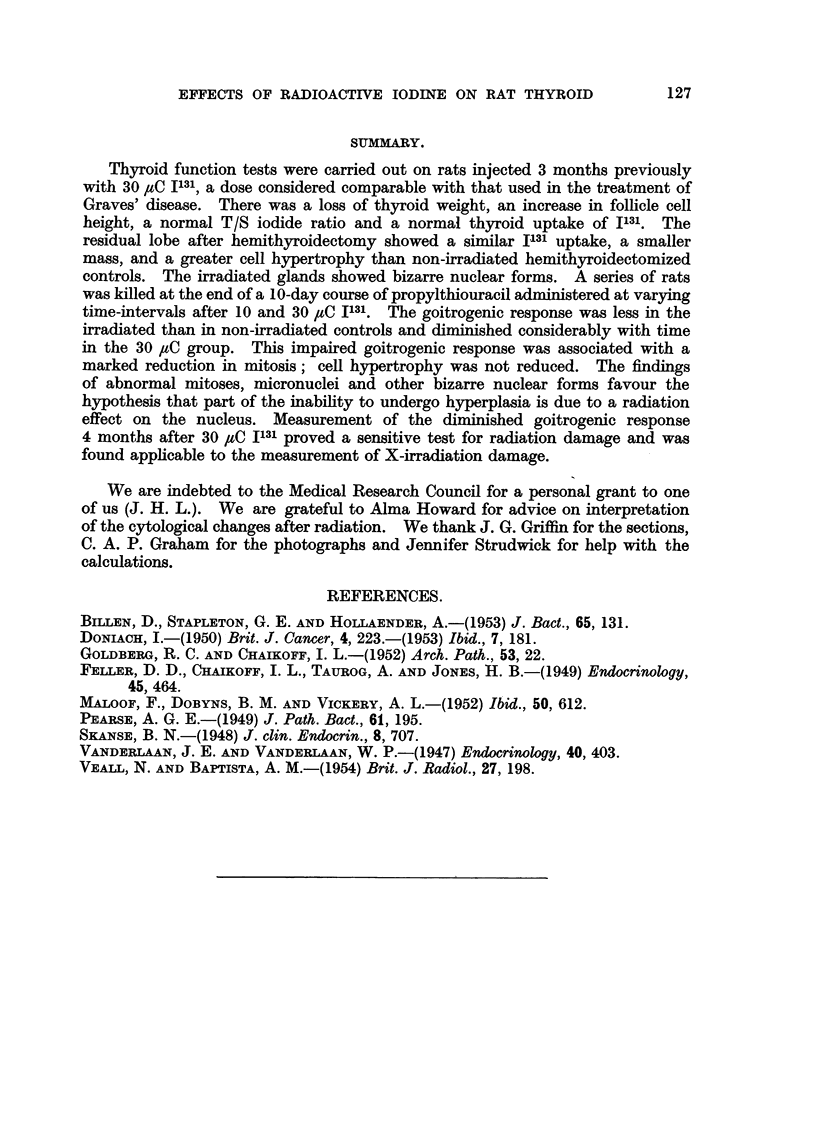


## References

[OCR_01119] BILLEN D., STAPLETON G. E., HOLLAENDER A. (1953). The effect of x-radiation on the respiration of Escherichia coli.. J Bacteriol.

[OCR_01120] DONIACH I. (1950). The effect of radioactive iodine alone and in combination with methylthiouracil and acetylaminofluorene upon tumour production in the rat's thyroid gland.. Br J Cancer.

[OCR_01124] FELLER D. D., CHAIKOFF I. L. (1949). The changes induced in iodine metabolism of the rat by internal radiation of its thyroid with I131.. Endocrinology.

[OCR_01128] MALOOF F., DOBYNS B. M., VICKERY A. L. (1952). The effects of various doses of radioactive iodine on the function and structure of the thyroid of the rat.. Endocrinology.

[OCR_01129] PEARSE A. G. E. (1949). The cytochemical demonstration of gonadotropic hormone in the human anterior hypophysis.. J Pathol Bacteriol.

[OCR_01133] VEALL N., BAPTISTA A. M. (1954). A multi-tube gamma counting apparatus for small samples.. Br J Radiol.

